# A Nomogram for Predicting Non-Rebound in HBV-Infected Pregnant Women With Mother-to-Child Transmission Prevention

**DOI:** 10.3389/fmed.2021.746759

**Published:** 2021-11-02

**Authors:** Chun-Rui Wang, Guo-Chao Zhong, Zhi-Wei Chen, Peng Hu

**Affiliations:** ^1^Department of Infectious Diseases, Key Laboratory of Molecular Biology for Infectious Diseases, Ministry of Education, Institute for Viral Hepatitis, The Second Affiliated Hospital, Chongqing Medical University, Chongqing, China; ^2^Department of Hepatobiliary Surgery, The Second Affiliated Hospital of Chongqing Medical University, Chongqing, China

**Keywords:** hepatitis B virus, pregnancy, nomogram, treatment cessation, virologic rebound

## Abstract

**Background:** Current guidelines recommend that pregnancies with mother-to-child transmission (MTCT) prevention can cease antiviral treatment after delivery. We aimed to develop a nomogram for predicting non-rebound in HBV-infected pregnant women with MTCT prevention after post-partum nucleos(t)ide analogs (NAs) withdrawal based on parameters before treatment cessation.

**Methods:** Pregnant women receiving antiviral therapy for MTCT prevention and who withdrew from taking NAs after delivery were included in this study. We used the least absolute shrinkage and selection operator (LASSO) logistics and a two-way stepwise regression to select prognostic factors for the risk model, and the concordance index (C-index) was used to assess its discrimination. Internal validation was performed through bootstrapping.

**Results:** Of 92 included patients, 16 and 76 experienced non-rebound and virologic rebound within 48 weeks of post-partum NAs cessation, respectively. Platelet to lymphocyte ratio (PLR) at 34 ± 2 weeks of gestation, a reduction in hepatitis B surface antigen (HBsAg) from baseline to 34 ± 2 weeks of gestation, and hepatitis B virus (HBV) DNA declining from baseline to the end of treatment (EOT) were entered into the final risk model. Its C-index was 0.91 (95% CI, 0.82–0.99), and it reached as high as 0.88 after bootstrapping validation. The decision curve and decision tree were further developed to facilitate the application of this model.

**Conclusions:** We developed a nomogram for predicting non-rebound in pregnant women with MTCT prevention after the withdrawal of antiviral agents, which facilitates physicians in making appropriate treatment recommendations.

## Introduction

Mother-to-child transmission (MTCT) remains a leading cause of hepatitis B virus (HBV) infection in China, accounting for 40–50% of new HBV infections (1). Women of childbearing age with chronic hepatitis B (CHB) infection were the primary sources of HBV infection through MTCT. The overall efficacy rate of combining hepatitis B vaccine with hepatitis B immunoglobulin as a preventive strategy ranges from 90 to 95%. However, 8–15% of pregnant women with a positive hepatitis B e antigen (HBeAg) and a high viral load fail to achieve prevention (2–4). Recent guidelines have proposed that antiviral prophylaxis during the second or third trimester of pregnancy is effective in preventing the MTCT of HBV. Pregnant women with CHB are generally young and are in the immune tolerance phase mostly. According to current guidelines, they are not optimal candidates for antiviral treatment. The purpose of nucleos(t)ide analogs (NAs) intervention during pregnancy is to reduce the risk of MTCT and to benefit the newborn. With the increased number of pregnant women treated with NAs, virology, and liver function abnormalities can be frequently observed in this population during pregnancy or postpartum. The reported rate of spontaneous alanine aminotransferase (ALT) flares ranges from 9 to 25%, with higher rates observed in the post-partum period than in pregnancy (5–7). The standard indications for HBV therapy and treatment duration have not been well defined and standardized (8–11), resulting in disorder clinical management.

Recently, hepatitis B core-related antigen (HBcrAg), virus pregenomic RNA (pgRNA), and hepatitis B core antibody have drawn clinicians' attention. As these new serological substitutes reflect a covalently closed circular DNA (cccDNA) activity, they are expected to be new serological markers for disease surveillance, antiviral efficacy evaluation, and recurrence prediction after drug withdrawal (12). Recent studies have found that HBV pgRNA is related to persistent HBV infection, HBV replication, and a rebound (13, 14). HBV pgRNA and HBcrAg levels have a great clinical significance in predicting the outcome of discontinuing treatment (15) but there is a lack of relevant reports among pregnant women.

Accordingly, we investigated the probability of non-rebound after treatment withdrawal and the association of common serum indicators (e.g., ALT level, HBeAg status, and HBV DNA load) and new serological substitutes (HBcrAg and pgRNA) with non-rebound after postpartum antiviral therapy in pregnancies with CHB. The primary objective of this study was to investigate the dynamic changes of serological indicators during pregnancy and to develop a nomogram for predicting non-rebound in pregnant women with MTCT prevention after post-partum NAs withdrawal based on post-cessation parameters. This study can provide a theoretical basis for clinicians to standardize the management of HBV MTCT.

## Materials and Methods

### Study Design and Patients

A retrospective cohort study was performed at a single center in Southwest China. Pregnant women with CHB infection undergoing treatment regularly in the Second Affiliated Hospital of Chongqing Medical University between January 2014 and December 2020 were evaluated for inclusion. Inclusion criteria for this study were based on the Chinese Guidelines for Prevention and Treatment of CHB (2019) (8). The study inclusion criteria were: (1) HBV infected pregnant women during the immune-tolerant phase characterized by HBV DNA ≥ 2 × 10^5^ IU/ml, normal ALT, and no obvious abnormality or mild inflammation in liver histology (8); (2) antiviral prophylaxis during pregnancy to prevent the MTCT of HBV; (3) regular follow-up until 48 weeks after delivery. Exclusion criteria were as follows: (1) patients receiving antiviral therapy for active hepatitis and (2) patients with other hepatotropic virus infections, HIV infection, liver cirrhosis, and pregnancy-related diseases (e.g., intrahepatic cholestasis during pregnancy). This study conforms strictly to the Ethical Guidelines of the 1975 Declaration of Helsinki. The study protocol was approved by the Ethics Committee of Second Affiliated Hospital of Chongqing Medical University.

### Follow-Up and Outcome Measures

Patients were followed up for 48 weeks after delivery until a virologic rebound, which was defined as a HBV DNA load increase over 2,000 IU/ml or an increase by >1log10 from the lowest level after treatment cessation (16). Pertinent information was recorded from medical records or by contacting the patient at the start of the treatment, which was defined as a baseline and referred to as 26 ± 2 weeks; at 8 weeks after treatment, which was defined as near delivery and referred to as 34 ± 2 weeks; at the treatment cessation, which was defined as the end of treatment (EOT) and referred to as post-partum 4 ± 2 weeks. Concurrently, serum was collected to measure HBcrAg and pgRNA levels.

### Predictive Variable Selection and Model Development

After inclusion, demographics characteristics, including age, parity status, and the type of antiviral therapy at baseline, were recorded. In addition, laboratory parameters, such as complete blood count, liver function test, and virological and serological markers, were collected at baseline, near delivery, and treatment cessation. We also calculated the neutrophil to lymphocyte ratio (NLR) and platelet to lymphocyte ratio (PLR) as they are important indicators of an immune response. For patients without a virological rebound after treatment withdrawal, the least absolute shrinkage and selection operator (LASSO) and logistic regression were used to identify risk factors. The LASSO regression analysis, a regression method that involves penalizing the absolute size of the regression coefficients, was used to select optimal predictors from post-cessation parameters. A two-way stepwise strategy was then performed in the multivariable logistics regression model to select independent predictors. Independent predictors obtained from the abovementioned screening progress were used to construct a nomogram. The concordance index (C-index) was used to evaluate the discrimination of the model and was validated by 1,000 bootstrap resampling, which resulted in a corrected C-index. Calibration curves were subsequently performed to assess the calibration of the model. We further developed a decision curve and decision tree to facilitate the application of this nomogram using R 3.6.1 software.

### Laboratory Methods

Routine biochemical assays were performed using commercially available autoanalyzers (Hitachi 7600-020, Tokyo, Japan), with a normal range of 0–40 U/L ALT. Serum HBV DNA levels were detected by Roche Amplicor/COBAS TaqMan HBV test v2.0 (Roche Molecular Diagnostics, Pleasanton, CA, USA) (detection limit, 100 IU/ml). Hepatitis serological markers were performed using enzyme-linked immunoassays. Serum HBcrAg was measured using a chemiluminescent immunoassay, Lumipulse G1200 automated analyzer (Fujirebio, Tokyo, Japan) with a lower quantification limit of 2 log U/ml. Serum pgRNA levels were measured using an ABI7500 quantitative real-time PCR system (ABI Laboratories, Natick, MA, USA), and the PCR kits were obtained from Sheng Xiang (Hunan, China) (the detection range from 2 × 10^2^ to 1 × 10^9^ copies/ml).

### Statistical Analysis

Continuous variables were expressed as median values [interquartile range (IQR)]. The Mann–Whitney *U* test and Chi-squared test were used for continuous and categorical variables, respectively. All statistical tests were two-sided, and the values of *p* < 0.05 were considered to be statistically significant.

## Results

### Clinical Characteristics of Patients and Outcomes

The flow chart of a process for identifying eligible patients is depicted in Supplementary Figure 1. A total of 146 pregnant women with HBV infection who were regularly followed up were evaluated, wherein 96 women received antiviral prophylaxis for the MTCT prevention of HBV and 92 women ceased the use of post-partum antiviral drugs. Of the 92 patients included in this study (median age: 28 years), 16 (17.4%) experienced a non-virologic rebound within 48 weeks of post-partum NA cessation, whereas 76 (82.6%) experienced a virologic rebound. The patients were classified into non-rebound and rebound groups according to HBV DNA load after post-partum antiviral drug cessation.

The baseline characteristics of 92 patients at 26 ± 2 weeks of gestation are described in Table 1. Clinical characteristics, including age, treatment, ALT level, HBeAg status, HBV DNA load, and hepatitis B surface antigen (HBsAg) level, were compared between the non-rebound and rebound group. Two groups showed no significant differences in age, treatment, times of MTCT prevention, infant's gender, and PLR (all *p* > 0.05). However, the median values of HBV DNA load, serum NLR, HBsAg, pgRNA, and HBcrAg levels in the rebound group were higher than those in the non-rebound group (all *p* < 0.05). Among 76 relapsed patients and 16 non-relapsed patients, 75 and 12 patients were positive for HBeAg, respectively (*p* = 0.003).

**Table 1:** Clinical characteristics of 92 recruited participants with MTCT prevention at baseline^a^.

**Characteristics**	**Overall**	**Rebound**	**Non-rebound**	***P***
Number of participants	92	76	16	–
Age (yeas)	28.0 (26.0–31.0)	28.0 (26.0–31.0)	28.0 (26.0–30.2)	0.714
Times of MTCT prevention, weeks	26 (24–28)	25 (24–28)	28 (26–28)	0.292
Infant's gender				0.137
Male, *n* (%)	42 (45.6%)	32 (42.1%)	10 (62.5%)	
Female, *n* (%)	50 (54.3%)	44 (57.9%)	6 (37.5%)	
Type of antiviral therapy				0.301
TDF, *n* (%)	41 (44.6%)	32 (42.1%)	9 (56.2%)	
LdT, *n* (%)	51 (55.4%)	44 (57.9%)	7 (43.8%)	
Follow-up time post-partum, months	15.0 (12.0–23.0)	15.0 (12.0–23.0)	16.0 (9.2–30.0)	0.352
HBeAg status at start of treatment				0.003
HBeAg-negative, *n* (%)	5 (5.4%)	1 (1.3%)	4 (25.0%)	
HBeAg-positive, *n* (%)	87 (94.6%)	75 (98.7%)	12 (75.0%)	
Serum log_10_HBV DNA, IU/mL	6.8 (6.5–7.3)	7.0 (6.6–7.4)	6.4 (5.6–6.8)	0.005
ALT, U/L	23.5 (15.8–34.2)	22.5 (14.6–34.0)	31.0 (20.2–39.5)	0.053
NLR	4.1 (3.6–5.3)	4.2 (3.7–5.5)	3.8 (3.3–4.4)	0.032
PLR	118.5 (94.0–135.5)	121.1 (99.4–139.4)	98.7 (89.0–120.7)	0.244
Serum log_10_HBsAg, IU/mL	4.4 (4.1–4.6)	4.5 (4.3–4.6)	3.7 (3.5–4.2)	<0.001
Serum log_10_HBeAg, PEIU/mL	3.1 (2.8–3.3)	3.2 (2.9–3.4)	2.4 (1.9–3.0)	<0.001
Serum log_10_pgRNA, copies/mL	7.8 (7.3–8.1)	7.8 (7.5–8.1)	6.6 (5.8–7.8)	0.010
Serum HBcrAg, logU/mL	8.6 (8.1–8.7)	8.6 (8.3–8.7)	7.8 (6.6–8.5)	0.008

*Continuous variables were expressed as median [interquartile range (IQR)], and categorical variables were expressed as counts (percentage).*

a *Baseline: at 26 ± 2 weeks of gestation, namely the time to start treatment.*

*MTCT, mother-to-child transmission; TDF, tenofovir disoproxil fumarate; LdT, telbivudine; HBV, hepatitis B virus; ALT, alanine aminotransferase; NLR, neutrophil to lymphocyte ratio; PLR, platelet to lymphocyte ratio; HBsAg, hepatitis B surface antigen; HBeAg, hepatitis B e antigen; pgRNA, pregenomic RNA; HBcrAg, hepatitis B core-related antigen*.

### Kinetics of Virological and Serological Indicators in Patients With MTCT Prevention After NAs Therapy

Near delivery, at 34 ± 2 weeks of gestation, we compared routine detection indicators and assessed the decline of these indicators. The results in Supplementary Table 1 show that there are significant differences in PLR, serum pgRNA, HBcrAg level, Δ HBV DNA, Δ HBsAg, and Δ pgRNA between the two groups (all *p* < 0.05). Specifically, the median levels of pgRNA, HBcrAg, and PLR of non-relapsed patients were significantly lower than those of relapsed patients [6.6 (IQR 5 0.0–7.9)] vs. 7.67 (IQR 7.1–8.0) log copies/ml, *p* = 0.042; 7.9 (IQR 6.4–8.4) vs. 8.5 (IQR 8.2–8.7) log U/ml, *p* = 0.025, and 100.9 (IQR 93.4–113.3) vs. 135.0 (IQR 98.8–177.1), *p* = 0.035, respectively. The decline of HBV DNA and HBsAg in the non-rebound group was lower than that in the rebound group, whereas the decline of pgRNA in the non-rebound group was higher than that in the rebound group.

At EOT of 4 ± 2 weeks post-partum, non-relapsed patients had lower HBeAg [2.8 (IQR 1.5, 3.1)] vs. 3 [(IQR 2.6, 3.3) log IU/ml, *p* = 0.047], HBcrAg [7.8 (5.9, 8.6) vs. 8.3 (7.3, 8.7) log U/ml, *p* = 0.005], and pgRNA levels [6.8 (4.2, 8.0) vs. 7.7 (6.6, 8.1) log_10_ copies/ml, *p* = 0.051)] (Supplementary Table 2) than relapsed patients. Likewise, non-relapsed patients showed a lower level of Δ HBV DNA and Δ HBsAg [2.6 (IQR 2.2–2.9) vs. 3.8 (IQR 3.3–4.3log_10_ IU/ml), *p* < 0.001]; [−0.5 (IQR −0.6–0.1)] vs. 0.0 (IQR −0.3–0.2) log_10_ IU/ml, *p* = 0.011], respectively. Both non-rebound and rebound groups showed significant suppression of a viral load at 34 ± 2 weeks of gestation and at the EOT. The median HBV DNA loads at 34 ± 2 weeks of gestation were 3.1 (IQR 3.0–4.4) in non-relapsed patients and 3.5 (IQR 3.0–4.2) log_10_ IU/ml in relapsed patients (*p* = 0.906). When antiviral therapy was ceased, the median HBV DNA loads were 3.8 (IQR 3.0–4.6) in non-relapsed patients and 3.0 (3.0–3.6) log_10_ IU/ml in relapsed patients (*p* = 0.008).

About 92 patients with MTCT prevention ceased drugs after delivery, of whom 16 experienced non-rebound and 76 experienced a virologic rebound. A detailed description is shown in Supplementary Table 3. The median level of HBV DNA loads and HBsAg were 3.0 (IQR 2.0, 3.8), 3.65 (IQR 3.51, 3.85) in non-relapsed patients and 6.81(IQR 6.14, 7.19) log_10_ IU/ml, 4.63(IQR 4.25, 4.80) log_10_ IU/ml in relapsed patients, respectively (*p* < 0.001).

### Construction of the Non-Rebound Prediction Based on Parameters Before NAs Cessation

#### The Predicted Risk Model Consisted of Three Selected Factors

A total of 36 variables described earlier, including HBV DNA, pgRNA, HBcrAg, ALT, HBsAg, HBeAg, NLR, PLR at baseline, near delivery, and at EOT, along with their declining values were entered into the LASSO logistics regression model for the selection of predictors. Eight variables with non-zero coefficients are shown in Supplementary Figure 2A. Supplementary Figure 2B shows the changes of LASSO coefficients with log λ. These eight predictors were subsequently screened in the multivariable logistics regression model using a two-way stepwise strategy (Table 2). Finally, three variables, including PLR at 34 ± 2 weeks of gestation (PLR34), a decline of HBsAg from baseline to 34 ± 2 weeks of gestation (Δlog_10_HBsAg34), and a decline of HBV DNA from baseline to EOT (ΔHBVDNAEOT), were determined as independent predictors for non-rebound. In addition, the C-index of the logistics regression model was 0.905 (95% CI, 0.824–0.986) and was corrected to 0.883 *via* bootstrapping validation.

**Table 2:** Factors predictive of post-partum HBV DNA non-rebound for 92 patients with MTCT prevention.

Variables^*^	**Univariable analysis**		**Multivariable analysis**	
	**OR (95% CI)**	***P*** **-Value**	**OR (95% CI)**	***P*** **-Value**
**At baseline**				
ALT, U/L	1.02 (0.99–1.05)	0.128		
Log_10_HBsAg, IU/mL	0.08 (0.02–0.32)	0.000		
NLR	0.57 (0.34–0.95)	0.037		
**At 34 ± 2 weeks of gestation**				
Log_10_pgRNA, Copies/mL	0.62 (0.40–0.92)	0.023		
PLR	0.98 (0.97–0.99)	0.037	0.97 (0.95–0.99)	0.011
Δlog_10_HBsAg^a^, IU/mL	0.07 (0.01–0.38)	0.002	0.07 (0.01–0.89)	0.040
**At the end of treatment**				
HBcrAg, log U/mL	0.66 (0.46–0.97)	0.028		
Δlog_10_HBVDNA^b^, IU/mL	0.31 (0.17–0.59)	0.000	0.23 (0.09–0.70)	0.005

* *The variables enrolled in the logistic regression analysis were continuous variables.*

a *Decline from baseline to near delivery.*

b *Decline from baseline to the end of treatment (EOT).*

*HBV, hepatitis B virus; ALT, alanine aminotransferase; NLR, neutrophil to lymphocyte ratio; PLR, platelet to lymphocyte ratio; HBsAg, hepatitis B surface antigen; pgRNA, pregenomic RNA; HBcrAg, hepatitis B core-related antigen*.

#### Prognostic Nomogram Based on Selected Predictors Showed Useful Clinical Application

**Figure 1:**
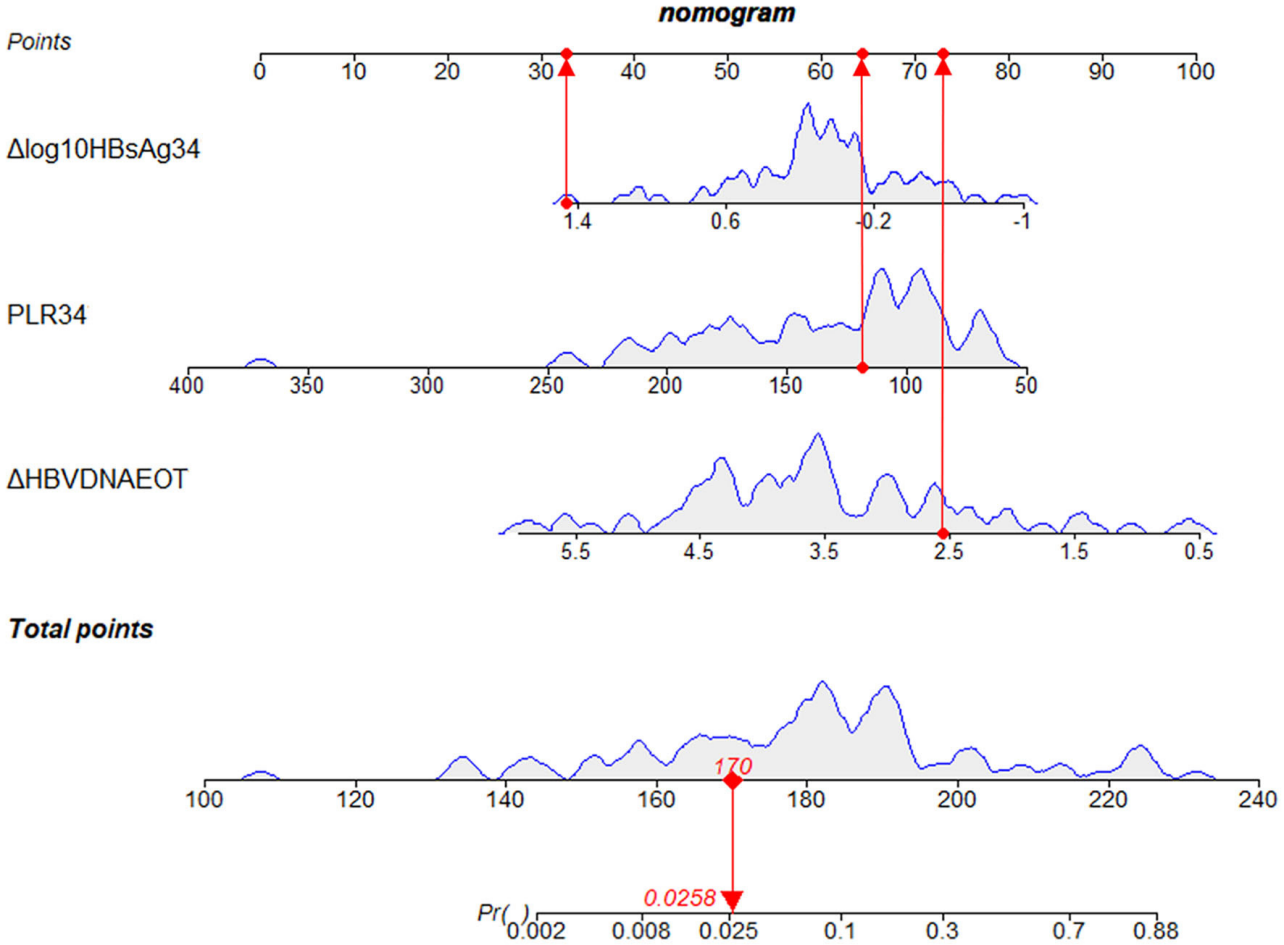
A nomogram to predict the probability of non-rebound outcomes in patients. We took a given patient as an example to explain the meaning of the nomogram. Specifically, the values of Δlog_10_HBsAg34, PLR34, and ΔHBVDNAEOT about the given patient were 1.3 log_10_ IU/ml, 118.64, and 2.56 log_10_ IU/ml, respectively. The corresponding scores on the “Points” scale were 33, 64, and 73, respectively, which were presented as red dots. By summing the points of each independent parameter, the total points projected on the bottom scales were 170, which was presented as a red diamond on the line, indicating that the probability of a non-rebound outcome was 2.58%. PLR34, PLR at 34 ± 2 weeks of gestation; Δlog_10_HBsAg34, a decline of HBsAg from baseline to 34 ± 2 weeks of gestation; ΔHBVDNAEOT, a decline of HBVDNA from baseline to the end of treatment; Pr, probability of a non-rebound outcome.

**Figure 2:**
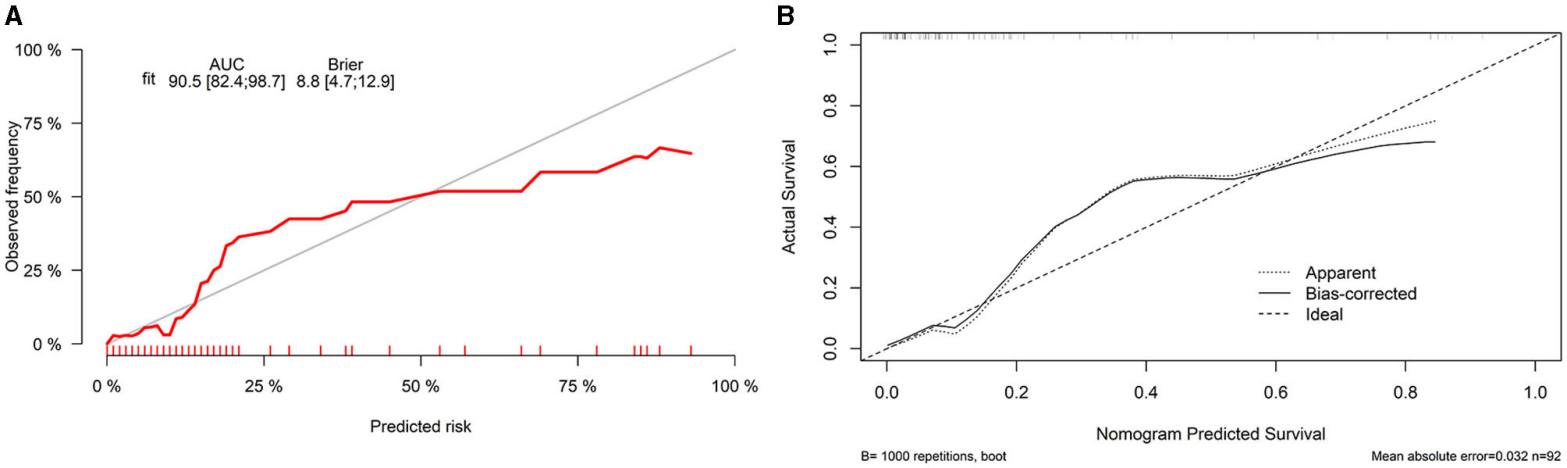
Discrimination and calibration of a nomogram. **(A)** Concordance index (C-index) and **(B)** calibration curve.

**Figure 3:**
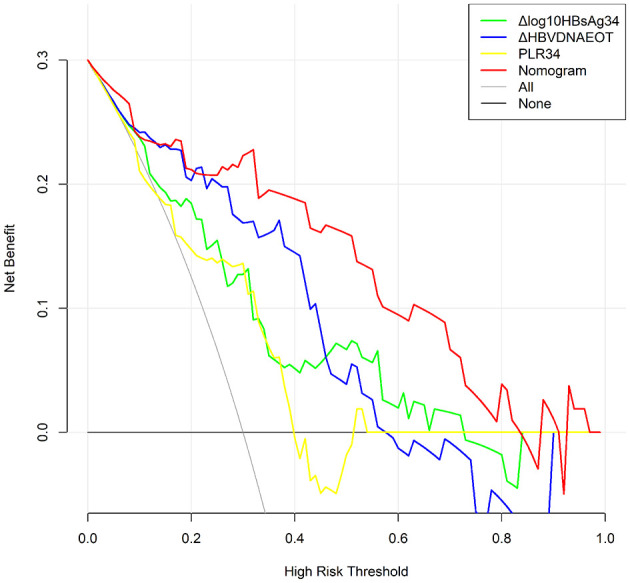
Decision curve for the non-rebound outcome predictive model. All, Presume that all patients do not rebound after treatment withdrawal. None, Presume that all patients rebound after treatment withdrawal. In the range of 0.3–0.8, the final complex model showed a higher net benefit rate than a single index. The clinical application was positively correlated with the area under the curve.

**Figure 4:**
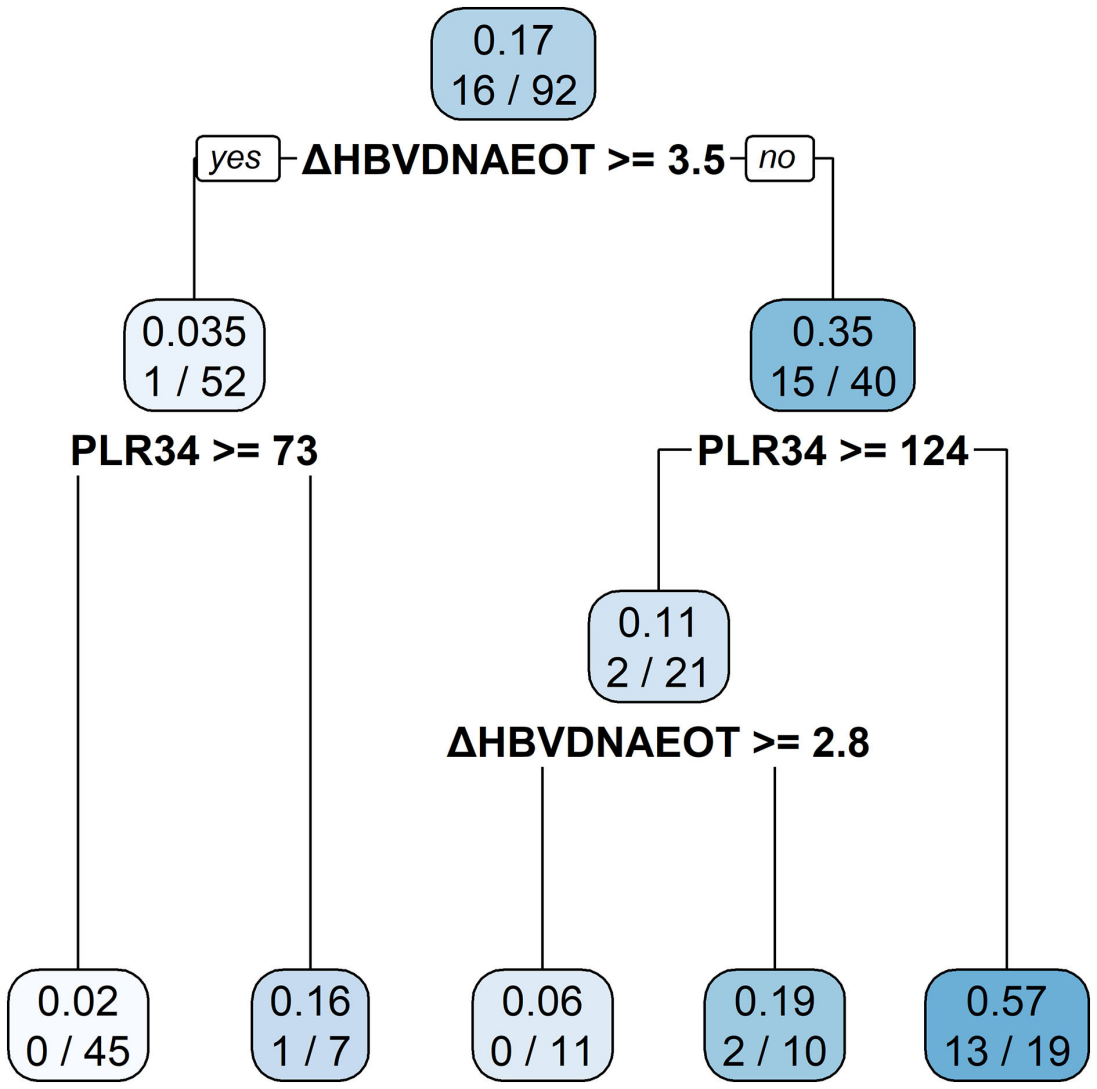
A decision tree for the non-rebound outcome predictive model. The decision tree consists of a root node, an internal node, and a leaf node, which are represented as squares. The darker the square, the higher probability of non-rebound will be presented.

Finally, we constructed a nomogram by combining prognostic factors, including PLR34, Δlog_10_HBsAg34, and ΔHBVDNAEOT (Figure 1). In the nomogram, the patient was scored according to the proportion of regression coefficient of each independent parameter. Each subtype of the variables corresponds to a point on the “Points” scale. By summing up the corresponding score, we could predict the probability of a non-rebound outcome. Then, we calculated the discrimination and calibration of this model. Its C-index was 0.91 (95% CI, 0.82–0.99) and reached as high as 0.88 after bootstrapping validation (Figure 2A). Then, we illustrated a calibration curve based on the actual incidence and prediction rate to assess our model, which showed that the curve had a similar predictive function compared to an ideal model (Figure 2B). In addition, compared with the intervention of all-or-none patients, the decision curve of this nomogram for predicting non-rebound revealed more net benefits if the threshold probability was <80%, which defines a high potential for clinical application (Figure 3). The decision tree developed for the clinician is shown in Figure 4. The decision paths with the highest expected utility values represented the optimal decisions.

## Discussion

To the best of our knowledge, this is the first cohort study of pregnant women with the MTCT prevention of HBV in Southwest China. The guidelines of HBV MTCT recommend that antiviral treatment could be withdrawn after delivery (17). The study included 92 patients, of whom 16 (17%) did not experience a virologic rebound after drug withdrawal, and evaluated potential factors in predicting non-rebound in patients with the MTCT prevention of HBV. Three independent predictors including PLR at 34 weeks of gestation, HBsAg reduction at 34 weeks of gestation, and a HBV DNA decline at post-partum treatment cessation, were identified and negatively correlated with non-rebound rates. The discrimination of the predicted model is 0.905, which could better distinguish the non-relapsed from a relapsed patient. We established a nomogram to accurately evaluate the prognosis of individuals and calculated the index node through a decision tree for the convenience of clinical decisions. Previous studies reported that the probability of post-partum non-rebound after drug withdrawal was 0–33% in pregnant women with HBV infection (18–21). The variability in rates may be related to the differences in rebound definitions, time of withdrawal, and study population. We defined a virologic rebound as a HBV DNA load increase over 2,000 IU/ml or an increase by >1 log 10 from the lowest level after treatment cessation. In previous studies of pregnancies with HBV infection, risk factors related to post-partum non-rebound after treatment cessation were not discussed. We first proposed the relationship between PLR and a virologic rebound in HBV-infected pregnant women. Hershko Klement et al. (22). investigated the kinetics of PLR during pregnancy and found that compared with the first and third trimester, the PLR value in the second trimester had the highest PLR, and the difference among the three stages was statistically significant. Our report lacked PLR data in the first trimester and showed no difference in PLR values between the second and third trimester, which could be related to the study population. PLR is defined as the ratio of platelets to lymphocytes, which plays an important role in the systemic inflammatory response and is closely related to the prognosis of viral hepatitis-related hepatocellular carcinoma (HCC), progression of HCV-related liver diseases, and early virologic response of CHB patients (23–25). Xu et al. (25) reported that PLR was positively correlated with a virologic response in treated CHB patients. Zhao et al. (26) retrospectively analyzed untreated CHB patients, HBV active carriers, and HBV-related compensated cirrhosis patients and demonstrated that PLR could reflect the levels of serum HBV DNA and HBeAg in CHB patients to some extent. PLR has not been reported in HBV-infected pregnant women.

In addition, this study suggested that a decline in HBsAg levels during late pregnancy was negatively correlated with non-rebound in our population. Different perspectives exist on the role of serum HBsAg in relation to antiviral therapy. Some scholars reported that HBsAg level at baseline or EOT, and its reduction during treatment could not effectively predict virologic rebound (27, 28). However, several studies showed that HBsAg levels at EOT were significantly associated with a rebound after the discontinuation of NAs. Lower HBsAg level, especially <300 IU/ml, was associated with a lower rebound rate after treatment withdrawal (29–31). Our investigation suggested that the HBsAg level in relapsed patients was higher than that of non-relapsed patients during follow-up, and a slower HBsAg reduction could predict higher non-rebound rates. In a previous study, we followed up 26 CHB patients treated with interferon for 48 weeks and found that HBsAg quantification after 12 weeks of treatment, as an independent predictor, was negatively correlated with HBeAg seroconversion (32). Likewise, the kinetics of HBV DNA load at EOT was negatively correlated with non-rebound rates in our study. As mentioned above, the probability of virologic rebound in HBV-infected pregnant women after drug withdrawal was 67–100%. Previous studies focused on the risk factors of a post-partum hepatitis flare. Liu et al. (18) reported that the rate of a post-partum hepatitis flare was positively correlated with HBV DNA load at baseline and delivery, whereas Kim et al. (20) believed that patients with elevated ALT before treatment had a higher incidence of post-partum hepatitis flare. The endpoint of our study was the non-rebound rates evident within 48 weeks after cessation in HBV-infected pregnant women. We found that the HBV DNA load in the non-rebound group was higher than that in the rebound group, which contradicts the results reported by Liu et al. This controversy could be explained by different definitions of endpoint and monitoring time.

Finally, we estimated the kinetics of HBcrAg and pgRNA levels at baseline, near delivery, and at EOT. We found that these two indicators in the non-rebound group were lower than those in the rebound group during follow-up, which is consistent with the results reported by Seto et al. (33). They assessed the serological parameters of 114 treated CHB patients and found that serum HBV RNA at EOT was an independent predictor of a viral rebound after treatment discontinuation. Rong et al. evaluated pgRNA levels after the cessation of NA treatment and concluded that the area under the receiver operating characteristic curve value of EOT HBV RNA was higher than that of HBV DNA plus HBsAg, which may be considered to predict clinical recurrence (34). PgRNA levels increased in patients with clinical recurrence after drug withdrawal and remained at a low level in patients without recurrence (35). Another study demonstrated that HBcrAg level > 3.7log10 IU/ml at EOT was correlated with a virologic rebound after cessation (36). The Japan Society of Hepatology suggested that it was possible to predict the risk of rebound based on HBsAg and HBcrAg levels at EOT and patients with HBsAg <80 IU/ml andHBcrAg < 3log10 U/ml at EOT had a lower risk of rebound (37). Based on these results, we conclude that HBcrAg and pgRNA levels could be represented as predictors of a viral rebound after drug withdrawal and could serve as a guide to clinical cessation.

Our study has some limitations. Firstly, the sample size was relatively small, and the follow-up time was insufficient to provide accurate data after cessation. Considering patient compliance and reports from previous studies, we selected a virologic rebound within 48 weeks as the primary endpoint. However, patients with recurrence after 48 weeks were not included in our analysis. Thus, the long-term clinical results after NA cessation need to be further evaluated in a study with a larger sample size. Secondly, the patients were enrolled from the outpatient department of the Second Affiliated Hospital of Chongqing Medical University, which might lead to selection bias. Finally, our predictive nomogram needs further external verification in different centers to check for the accuracy and applicability of our results.

In conclusion, we identified three independent risk factors, namely PLR and HBsAg reduction at 34 weeks of gestation, and HBV DNA decline at EOT, which were negatively correlated with the rates of non-rebound. The predicted nomogram was further constructed to facilitate physicians in making appropriate treatment recommendations.

## Data Availability Statement

The original contributions presented in the study are included in the article/Supplementary Material, further inquiries can be directed to the corresponding author/s.

## Ethics Statement

Our study protocol was reviewed and approved by the Ethics Committee of Second Affiliated Hospital of Chongqing Medical University. Written informed consent was obtained from the individual(s) for the publication of any potentially identifiable images or data included in this article.

## Author Contributions

C-RW and PH developed the hypothesis, study design and concept, and all authors made useful suggestions for study design and concept. C-RW completed data collection, analysis, and interpretation. G-CZ and Z-WC participated in the follow-up of patients. All authors contributed to the article and approved the submitted version.

## Funding

This work was supported in part by the National Natural Science Foundation of China (81772171) and the National Science and Technology Major Project of China (2017ZX10202203-007, 2017ZX10202203-008, and 2018ZX10302-206-003).

## Conflict of Interest

The authors declare that the research was conducted in the absence of any commercial or financial relationships that could be construed as a potential conflict of interest.

## Publisher's Note

All claims expressed in this article are solely those of the authors and do not necessarily represent those of their affiliated organizations, or those of the publisher, the editors and the reviewers. Any product that may be evaluated in this article, or claim that may be made by its manufacturer, is not guaranteed or endorsed by the publisher.
