# Supplementary material for: A Nomogram for Predicting Non-Rebound in HBV-Infected Pregnant Women With Mother-to-Child Transmission Prevention
Source: Front Med (Lausanne). 2021 Nov 2;8:746759. doi: 10.3389/fmed.2021.746759 (PMC8596549; doi:10.3389/fmed.2021.746759)
Supplement: Supplementary file 1 [file Data_Sheet_1.docx]

Supplementary Material

# Supplementary Figures and Tables

## Supplementary Figures

**
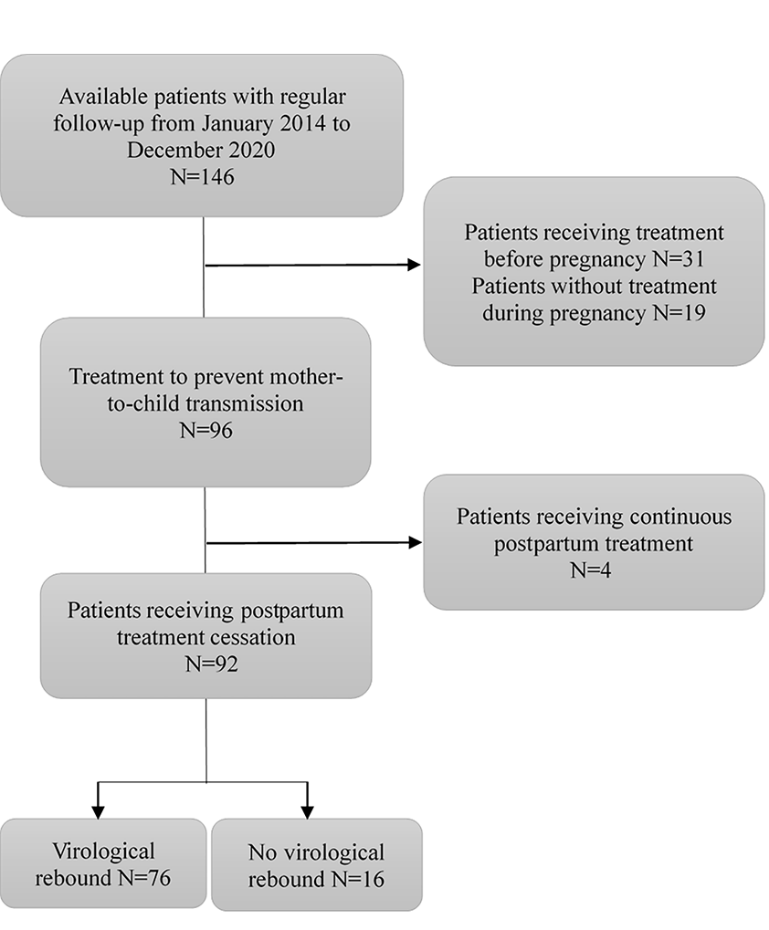
**

**Supplementary Figure 1.** The flow chart of identifying eligible patients.


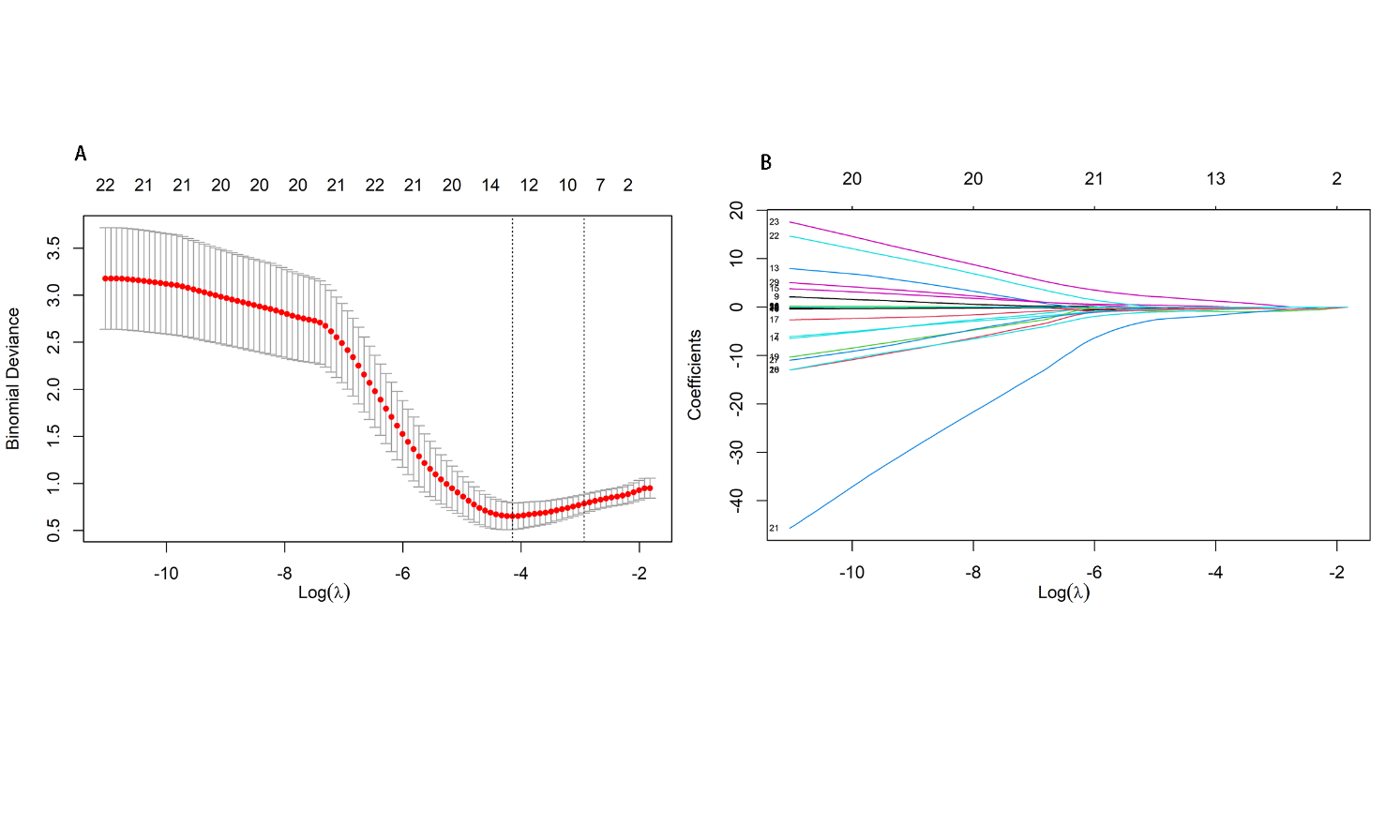


**Supplementary Figure 2.** LASSO logistics regression plot. (A) Plot of partial likelihood deviance; (B) plot of LASSO coefficient profiles.

## Supplementary tables

| **Supplementary Table 1.** Kinetics of virologic and serological indicators in patients with MTCT prevention near delivery^*^ | | | | |
| --- | --- | --- | --- | --- |
| Characteristics | Overall | Rebound | Non-rebound | *P* |
| Number of participants | 92 | 76 | 16 | - |
| Log_10_HBV DNA, IU/ml | 3.4 (3.0-4.2) | 3.5 (3.0-4.2) | 3.1 (3.0-4.4) | 0.906 |
| ΔLog_10_HBV DNA, IU/ml | 3.4 (2.5-4.0) | 3.5 (2.8-4.0) | 2.4 (2.1-3.6) | 0.037 |
| ALT(U/L) | 21.5 (15.0-32.0) | 20.0 (14.0-31.0) | 30.0 (19.5-34.2) | 0.890 |
| NLR | 4.6 (3.5-6.0) | 4.7 (3.7-6.5) | 4.3 (3.2-4.9) | 0.716 |
| PLR | 122.9 (96.7-170.0) | 135.0 (98.8-177.1) | 100.9 (93.4-113.3) | 0.035 |
| Serum log_10_HBsAg, IU/mL | 4.3 (4.1-4.5) | 4.4 (4.1-4.6) | 4.1 (3.6-4.4) | 0.388 |
| Serum log_10_HBeAg, PEIU/mL | 3.0 (2.5-3.2) | 3.0 (2.5-3.3) | 2.6 (1.9-3.1) | 0.134 |
| ΔSerum log_10_HBsAg, IU/mL | 0.1 (-0.1-0.3) | 0.1 (-0.1-0.3) | -0.1 (-0.5-0.0) | <0.001 |
| ΔSerum log_10_HBeAg, PEIU/mL | 0.1 (-0.1-0.3) | 0.1 (-0.1-0.3) | -0.1 (-0.6-0.4) | 0.189 |
| Serum log_10_pgRNA, Copies/mL | 7.6 (6.9-8.0) | 7.7 (7.1-8.0) | 7.2 (5.6-7.9) | 0.014 |
| Serum HBcrAg, log U/mL | 8.4 (7.9-8.7) | 8.5 (8.1-8.7) | 7.9 (6.7-8.4) | 0.045 |
| ΔSerum log_10_pgRNA, Copies/mL | 0.1 (-0.3-0.6) | 0.1 (-0.2-0.6) | 0.2 (-0.7-0.6) | 0.897 |
| ΔSerum HBcrAg, log U/mL | 0.1 (-0.1-0.4) | 0.1 (-0.1-0.4) | 0.0 (-1.2-0.4) | 0.630 |

*Continuous variables were expressed as median (interquartile range). ^*^Near delivery: at (34±2) weeks of pregnancy, namely 2 months after treatment Δ: Decline from baseline to near delivery. Abbreviations: MTCT: mother-to-child transmission; DNA, deoxyribonucleic acid; HBV, hepatitis B virus; ALT, alanine aminotransferase; NLR, neutrophil to lymphocyte ratio; PLR, platelet to lymphocyte ratio; HBsAg, hepatitis B surface antigen; HBeAg, hepatitis B e antigen; pgRNA, pregenomic RNA; HBcrAg, hepatitis B core-related antigen;*

| **Supplementary Table 2.** Kinetics of virologic and serological indicators in patients with MTCT prevention at the end of treatment | | | | |
| --- | --- | --- | --- | --- |
| Characteristics | Overall | Rebound | Non-rebound | *P* |
| Number of participants | 92 | 76 | 16 | - |
| Serum log_10_HBV DNA, IU/mL | 3.0 (3.0-3.8) | 3.0 (3.0-3.6) | 3.8 (3.0-4.6) | 0.008 |
| Δ log_10_HBV DNA, IU/mL | 3.6 (2.9-4.3) | 3.8 (3.3-4.3) | 2.6 (2.2-2.9) | <0.001 |
| ALT, U/L | 33.1 (25.0-56.8) | 32.5 (24.8-58.3) | 41.5 (27.0-56.6) | 0.835 |
| NLR | 2.0 (1.5-2.5) | 2.0 (1.5-2.5) | 2.1 (1.4-2.8) | 0.643 |
| PLR | 121.3 (98.0-149.1) | 121.4 (98.1-151.9) | 119.6 (96.7-136.0) | 0.725 |
| Serum log_10_HBsAg, IU/mL | 4.5 (4.1-4.7) | 4.5 (4.2-4.7) | 4.2 (4.0-4.7) | 0.256 |
| Serum log_10_HBeAg, PEIU/mL | 3.0 (2.5-3.3) | 3.0 (2.6-3.3) | 2.8 (1.5-3.1) | 0.029 |
| Δlog_10_HBsAg, IU/mL | -0.1 (-0.4-0.1) | 0.0 (-0.3-0.2) | -0.5 (-0.6-0.1) | 0.011 |
| Δlog_10_HBeAg, PEIU/mL | 0.1 (-0.4-0.3) | 0.1 (-0.2-0.3) | -0.3 (-0.7-0.6) | 0.537 |
| Serum log_10_pgRNA, Copies/mL | 7.7 (6.1-8.1) | 7.7 (6.6-8.1) | 6.8 (4.2-8.0) | 0.019 |
| Serum HBcrAg, log U/mL | 8.3 (7.2-8.6) | 8.3 (7.3-8.7) | 7.8 (5.9-8.6) | 0.020 |
| ΔSerum log_10_pgRNA, Copies/mL | 0.1(-0.3-1.3) | 0.0 (-0.3-1.2) | 0.3 (-0.2-0.6) | 0.547 |
| ΔSerum HBcrAg, log U/mL | 0.1 (-0.1-1.3) | 0.1 (-0.1-1.2) | 0.2 (-0.7-2.1) | 0.899 |

*Continuous variables expressed in median (interquartile range). Δ: Decline from baseline to the end of treatment. Abbreviations: MTCT: mother-to-child transmission; DNA, deoxyribonucleic acid; HBV, hepatitis B virus; ALT, alanine aminotransferase; NLR, neutrophil to lymphocyte ratio; PLR, platelet to lymphocyte ratio; HBsAg, hepatitis B surface antigen; HBeAg, hepatitis B e antigen; pgRNA, pregenomic RNA; HBcrAg, hepatitis B core-related antigen;*

| **Supplementary Table 3.** Kinetics of virologic and serological indicators in patients with MTCT prevention after treatment cessation | | | | |
| --- | --- | --- | --- | --- |
| Characteristics | Overall | Rebound | Non-rebound | *P* |
| Number of participants | 92 | 76 | 16 | - |
| Serum log_10_HBV DNA, IU/mL | 7.1 (6.4-7.4) | 7.1 (6.7-7.4) | 3.1 (3.0-3.6) | <0.001 |
| ALT, U/L | 38.0 (25.0-66.0) | 36.0 (26.0-85.0) | 38.0 (20.0-48.8) | 0.171 |
| NLR | 1.8 (1.4-2.2) | 1.9 (1.5-2.6) | 1.4 (1.1-1.8) | 0.233 |
| PLR | 117.2 (92.5-154.0) | 120.3 (95.8-158.3) | 98.8 (89.7-117.3) | 0.425 |
| Serum log_10_HBsAg, IU/mL | 4.4 (3.9-4.8) | 4.6 (4.2-4.8) | 3.7 (3.6-4.0) | 0.003 |
| Serum log_10_HBeAg, PEIU/mL | 3.2 (2.5-3.3) | 3.2 (3.0-3.3) | 0.0 (0.0-1.6) | <0.001 |

*Continuous variables expressed in median (interquartile range). Abbreviations: MTCT: mother-to-child transmission; DNA, deoxyribonucleic acid; HBV, hepatitis B virus; ALT, alanine aminotransferase; NLR, neutrophil to lymphocyte ratio; PLR, platelet to lymphocyte ratio; HBsAg, hepatitis B surface antigen; HBeAg, hepatitis B e antigen; pgRNA, pregenomic RNA; HBcrAg, hepatitis B core-related antigen;*
